# FDG and Non-FDG Radiopharmaceuticals for PET Imaging in Invasive Lobular Breast Carcinoma

**DOI:** 10.3390/biomedicines11051350

**Published:** 2023-05-03

**Authors:** Laura Gilardi, Lighea Simona Airò Farulla, Giuseppe Curigliano, Giovanni Corso, Maria Cristina Leonardi, Francesco Ceci

**Affiliations:** 1 Division of Nuclear Medicine, IEO European Institute of Oncology IRCCS, 20141 Milan, Italy; 2 Department of Oncology and Hemato-Oncology, University of Milan, 20122 Milan, Italy; 3 Division of New Drugs and Early Drug Development, IEO European Institute of Oncology IRCCS, 20141 Milan, Italy; 4 Division of Breast Surgery, IEO European Institute of Oncology, IRCCS, 20141 Milan, Italy; 5 European Cancer Prevention Organization (ECP), 20122 Milan, Italy; 6 Division of Radiotherapy, IEO European Institute of Oncology IRCCS, 20141 Milan, Italy

**Keywords:** PET/CT, lobular breast carcinoma, FDG, FES, FAPi

## Abstract

Invasive lobular cancer (ILC) is the second most frequent histological type of breast cancer (BC) and includes a heterogeneous spectrum of diseases with unique characteristics, especially the infiltrative growth pattern and metastatic spread. [^18^F]fluoro-2-deoxy-D-glucose positron emission tomography/computed tomography (FDG-PET/CT) is extensively used in oncology and BC patient evaluation. Its role in ILCs is considered suboptimal due to its low FDG avidity. Therefore, ILCs could benefit from molecular imaging with non-FDG tracers that target other specific pathways, contributing to precision medicine. This narrative review aims to summarize the current literature on the use of FDG-PET/CT in ILC and to discuss future opportunities given by the development of innovative non-FDG radiotracers.

## 1. Introduction

With more than two million cases registered worldwide, breast cancer (BC) is the most diagnosed cancer and the leading cause of cancer-related death in women [1]. The two main histological types of BC are invasive ductal carcinoma (IDC) and invasive lobular carcinoma (ILC), the latter accounting for 5–15% of all cases [2].

In the era of personalized medicine, ILC is increasingly emerging as a specific morpho-molecular BC entity with relevant differences in the transcriptomic profiles, clinical behavior, and metastatic pattern compared to IDC, which leads to notable implications on diagnosis and treatment [3].

ILC is characterized by small, round cells that infiltrate breast stroma and adipose tissue in a “single-file” fashion with no or minimal stromal fibrous reaction [4]. The infiltrative growth pattern leads to a more challenging radiographic detection for mammography and ultrasound [5]. Furthermore, the low grade and the hormone receptor positivity of most ILCs resulted in a sub-optimal diagnostic accuracy for [^18^F]fluoro-2-deoxy-D-glucose positron emission tomography/computed tomography (FDG-PET/CT) as well [6]. However, PET imaging can investigate other specific BC pathways [7] and could lead to additional, useful information in ILC.

**Figure 1:**
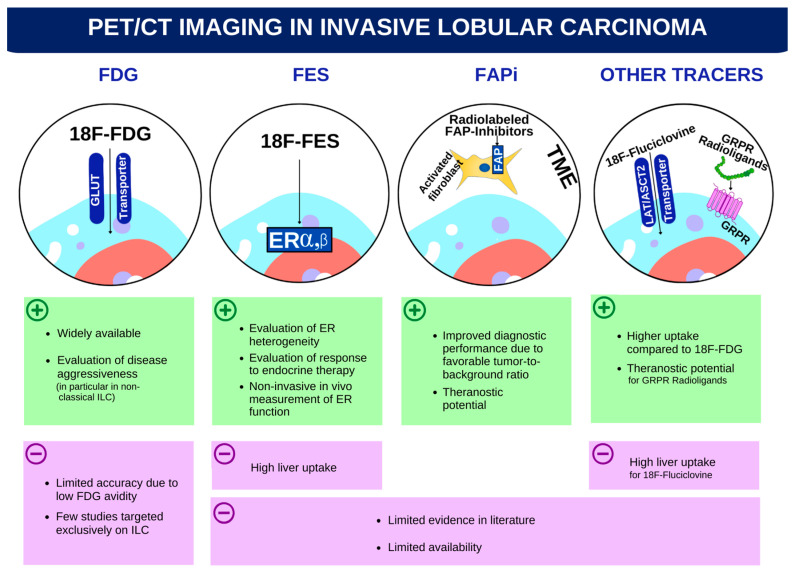
Strengths and limitations of PET radiopharmaceuticals for invasive lobular cancer imaging.

Our narrative review aims to summarize the current evidence about the use of FDG-PET in ILC and to explore future perspectives in non-FDG radiopharmaceuticals (Figure 1).

## 2. Invasive Lobular Breast Carcinoma: A Special Spectrum of Tumors

### 2.1. Histology and Molecular Characteristics

ILC is a histological subtype of BC with unique clinical and molecular features still largely unexplored. ILC gathers several histological patterns sharing the characteristic of lack of cell-to-cell adhesion related to loss of E-cadherin expression [8]. E-cadherin is a transmembrane calcium-dependent glycoprotein that plays an indispensable role in cell-to-cell adhesion and comprises extracellular, juxtamembrane, and intracellular domains. The intracellular domain interacts with α-, β-, γ-, and p120 catenins along with vinculin and establishes a complex of proteins with close interaction with the cytoskeleton. The loss of E-cadherin in ILC also results in the loss of α-, β-, and γ-catenins, while p120 catenin, which in normal breast epithelium is expressed on the cell membrane, is upregulated and relocated to the cytoplasm. These characteristics increase the tissue invasion by ILC cells [9]. E-cadherin dysregulation results from somatic mutations in the CDH1 gene on chromosome 16q22.1 (about 60% of cases) as well as by loss of heterozygosity at the CDH1 locus (>90% of cases) or both [2].

Other specific markers for ILC include cyclin D1 (80% of cases), cathepsin D (86%), Bcl-2 (89%), and GCDFP-15 (demonstrated in up to 90% of pleomorphic and signet ring subtypes) [10].

Classic ILC, which accounts for approximately half of all ILC cases, is characterized by small, relatively uniform cells that infiltrate the stroma in chain-like cords (the so-called “single-file” growth pattern) with limited inflammatory response and without significant breast tissue architecture. Non-classic forms are distinguished by morphology (solid, alveolar, trabecular, dispersed, and mixed) and cytology (pleomorphic, apocrine, histyocitoid, signet ring, and tubulolobular). The solid variant displays sheets of uniform cells with a high frequency of mitoses, while in the alveolar type, cells are organized in globular arrangements. The most aggressive pleomorphic variant exhibits a higher degree of cellular atypia, mitotic activity, and nuclear pleomorphism. After the classical type, mixed morphology represents the most common presentation [4,11].

Most ILCs have histological grades 1 and 2, and more than 90% express estrogen receptor (ER), while human epidermal growth factor receptor 2 (HER2) overexpression is rare (demonstrated in 3–5% of the classic type). On the contrary, the pleomorphic variant exhibits a higher proliferation index, lowest rates of ER positivity, and HER2 amplification in 35–80% of cases. Regarding the intrinsic molecular subtypes, it was observed in a high percentage of Luminal A and Luminal B tumors, while basal-like and HER2-enriched subtypes were less expressed [2,10,11].

### 2.2. Clinical Presentation and Metastatic Pattern

Compared with IDC, ILC is associated with older age, more advanced stage at presentation, and often multi-focal or multicentric disease. Despite the early advantage in disease-free and overall survival, longer-term follow-up showed more late relapses in ILC patients, frequently occurring more than 10 years after the initial diagnosis [10,12].

Another significant difference between IDC and ILC is the distant metastatic pattern. Although both can metastasize to common sites such as the liver, lung, and bone, ILC involves more frequently unusual sites such as the peritoneum, retroperitoneum, gastrointestinal tract, gynecologic organs, and leptomeninges. When ILC metastasizes to the gastrointestinal tract, the stomach, and small bowel are the most commonly involved, followed by the colon and rectum, but any gastrointestinal tract can be involved, from tongue to anus. Clinical presentations are predominantly nonspecific with abdominal pain, inappetence, nausea, and diarrhea and can progress to vomiting and bleeding in advanced cases. As for primary tumors, metastases tend to infiltrate affected organs in a diffuse manner, making assessment difficult both for radiological and nuclear medicine imaging [13,14].

### 2.3. Surgical Treatment

#### 2.3.1. Breast-Conserving Surgery

Although the literature reports less indication for breast-conserving surgery in the treatment of lobular breast cancer [15], this surgical approach is clearly indicated also in case of invasive or in situ pattern (LCIS). Breast magnetic resonance (MR) is mandatory prior to surgery to evaluate the presence of bilateral or multi-focal disease. In the specific case of a unifocal lesion or in the case of a multi-focal lesion in the same breast quadrant, conserving surgery is considered a safe procedure [16]. Breast margins evaluation after surgery is mandatory. The St. Gallen guidelines recommend “no ink on tumor” margins for women undergoing breast-conserving surgery, regardless of the presence of unifocal or multi-focal disease [17]. In the instance of focally positive margins at breast-conserving surgery, re-excision is strongly recommended, especially when the extent of margin involvement is anything beyond truly minimal.

#### 2.3.2. Mastectomy

Nipple-sparing mastectomy plus breast reconstruction is indicated in the case of multicentric lobular BC, when the breast size cup is small and when the nipple is not involved by tumor cells at the intra-operative examination [18]. In both prophylactic and therapeutic settings, nipple-sparing mastectomy currently represents a well-tolerated approach and, thanks to immediate breast reconstruction, an oncological treatment that respects a woman’s body image, physical and psychological well-being as well as the cosmetic outcome [19]. Improvements in surgical techniques such as robotic surgery [20] constituted novel notable innovations, strengthening and extending its use. Moreover, nipple-sparing mastectomy is acquiring an interesting role as a prophylactic tool for BC patients with CDH1 mutation and a significant family history of BC aggregation. Recent studies have indeed reported a relevant association between germline CDH1 germline mutations and lobular BC [21].

#### 2.3.3. Axillary Surgery

Sentinel node biopsy (SNB) is the standard approach for patients presenting with a clinically negative axilla and undergoing breast-conserving surgery. In cases of 1–2 positive sentinel node biopsies, completion of axillary dissection is not indicated when patients will receive post-lumpectomy radiation therapy and appropriate systemic adjuvant therapy [22]. Patients with residual, clinically positive nodes after neoadjuvant treatments are advised to have a completion axillary dissection. SNB is a safe procedure in cases of patients presenting with clinically positive (cN1) axillary nodes and who received neoadjuvant therapies downstaging to cN0. In cases of women with the residual nodal disease after neoadjuvant therapies on SNB, completion axillary dissection is generally warranted, even in the case of micrometastatic residual cancer at SNB [23,24].

### 2.4. Radiotherapy

The safety of breast conservative treatment versus mastectomy for ILC carcinoma has long been debated. Due to the higher incidence of diffuse and multi-focal lesions, generally undetectable at imaging, concerns were raised regarding the efficacy of whole breast irradiation in the presence of a potentially greater-than-expected residual tumor burden after breast-conserving surgery [25].

Currently, the literature indicates no difference in local relapse and survival outcomes for ILC receiving breast conservative surgery with free margins compared to IDC [26,27,28]. Schedules and techniques of adjuvant radiotherapy do not differ, considering histology and hypofractionation can be safely applied to ILC [29]. More aggressive histologic subtypes, such as the pleomorphic and signet ring variants, are associated with an increased rate of recurrence and poor prognosis compared to classic ILC, but it does not warrant a different therapeutic approach, including radiotherapy [30]. Regarding the use of a boost dose to the tumor bed, ILC, for its diffuse and multi-focal nature, is characterized by a higher potential residual burden, as shown by the higher rate of positive margins following breast-conserving surgery compared to IDC [31]. The SSO/ASTRO consensus on surgical margins status stated that there is no need to increase the boost dose in case of ILC as long as negative margins are obtained [32].

ILC was not associated with a higher rate of locoregional recurrence or decreased survival in clinically node-negative patients receiving SNB without axillary dissection in the presence of 1 or 2 node metastases [22], supporting the omission of any form of axillary radiotherapy. However, the radiation approach to the undissected axilla can vary across the centers. Higher rates of axillary nodal metastases for ILC compared to IDC have been observed, partly due to, on average, larger tumor size and more advanced stage [33]. Many nomograms predicting the probability of positive non-sentinel nodes incorporated ILC as a negative prognostic factor [34,35,36].

In clinical practice, many institutions [37,38] considered other variables than histology to design the radiation fields, such as tumor biology, extracapsular extension, micrometastases size, and younger age. Regarding partial breast irradiation (APBI), despite the common Luminal A-like profile of ILC, the typical multicentric aspect makes it a subject of controversy for its eligibility for APBI. Since this modality is confined to the tumor bed while sparing the remaining breast, the success of APBI lies in the selection of tumors with a low probability of having distant additional neoplastic foci. Following a more conservative approach, the ASTRO and ESTRO guidelines for APBI excluded ILC from the ideal candidates [39,40]. Conversely, the American Brachytherapy Society considered all the histologies eligible for APBI without distinction, based on the assumption that prognosis does not depend so much on the type of treatment as on tumor biology [41]. Recently, some phase III trials proved that APBI in ILC had similar outcomes compared to IDC [42]. In this context, breast MRI can be useful to improve patient selection for APBI [43]. A retrospective comparative analysis between ILC and IDC treated with intraoperative radiotherapy with electrons (IOERT) as APBI demonstrated a higher local relapse for ILC compared to IDC. Interestingly, using a propensity-score matching Luminal A ILC subtype presented a higher rate of “true” local relapse, occurring near or at the primary tumor bed, compared to IDC. A smaller IOERT collimator size was a statistically significant negative predictor factor, pointing out the importance of a proper radiation field extension to deal with neoplastic foci adjacent to the site of the primary tumor [44].

### 2.5. Medical Treatment of Invasive Lobular Cancer in the Adjuvant Setting

Treatment decisions for ILC patients are often derived from data on IDC patients, leading to possible divergencies in patient management across different institutions. In particular, the role of adjuvant chemotherapy (aCT) for patients with localized lobular BC (ILC) is still controversial. It is unclear what is the magnitude of the benefit of the CT in this setting. In the systematic review and meta-analysis of 38,387 patients across eight clinical studies, aCT was not associated with an improvement of OS (SHR 0.99; 95%CI 0.86–1.14), with low heterogeneity (I^2^ = 28%) and no publication bias (*p* = 0.43) [45]. The conclusions remained unchanged even after the sensitivity analysis. Coherently with the OS results, the value of aCT in improving DFS was unconfirmed, and as a result, a definite role of aCT for patients with ILC was not confirmed as well. However, as research gaps have been identified, prospective, controlled ad hoc investigations will be needed [45]. One of the most controversial decision points in the management of ILC is the indication for adjuvant chemotherapy, as ILC commonly presents estrogen receptor (ER)-positive, low proliferating, and well differentiated. In patients who have advanced or bilateral ILC, there is the possibility of administering neoadjuvant chemotherapy (NACT) both to assess in vivo sensitivity to different agents and to pursue downstaging. To date, the published evidence on gene signature in ILC is only available for OncotypeDX, a clinically validated assay for risk assessment of distant recurrence and prediction of chemotherapy benefit in patients with early-stage BC [46]. Using OncotypeDX to study ILC, four observational studies showed a distribution (based on the risk of recurrence score, ROR: 0–100) of lobular tumors in the low or intermediate-risk groups: 1.3–8% of cases were in the high-risk class (ROR > 25). Grade 1 and 2 ILCs almost all had low and intermediate ROR, while more than 10% of grade 3 tumors had high ROR. A study was conducted in order to understand the prognostic implications based on the Surveillance, Epidemiology, and End Results (SEER) dataset (n = 7316 patients), and it reported, for ILC patients with low and intermediate ROR, a BC-specific 5-year OS of 99%, while it was mildly worse for those with high ROR (96% OS) [47]. In patients with high ROR, a tendency to prescribe chemotherapy has been demonstrated, consistent with the current clinical use of gene signatures to guide the indication for ACT; despite this, no improvement in OS was associated with patients with high ROR treated with chemotherapy.

In postmenopausal ILC patients, the indications for adjuvant use of endocrine therapy are mainly based on the subgroup analysis of two randomized, controlled, phase 3 clinical trials: the MA 27 trial (comparing the two aromatase inhibitors exemestane and anastrozole) and the TEAM trial (comparing adjuvant use of tamoxifen followed by exemestane sequentially vs. exemestane for five years) [48]. However, the TEAM study regarding relapse-free survival for ILC patients taking an aromatase inhibitor for five years or shorter sequentially to tamoxifen showed a similar result. The ER-poor subgroup (Allred score < 7) seemed to have greater benefits from the sequential strategy [49].

For endocrine-positive BC, clinical options have been implemented by clinical development and adoption of cyclin-dependent kinase (CDK) 4 and 6 inhibitors [50]. A pooled analysis by the American Food and Drug Administration (FDA) reported a benefit of escalating endocrine therapy with CDK4/6 inhibitors in 269 ILC patients, with improved median progression-free survival up to twelve months (hazard ratio 0.59), compared with endocrine therapy alone [51]. Several studies are testing the addition of anti-CDK4/6 compounds to standard endocrine therapies, either as risk-adapted escalation therapy in the post-neoadjuvant setting (e.g., PENELOPE-B) or as an adjuvant strategy (e.g., PALLAS), although not designed only for ILC patients [52].

## 3. PET Imaging in Breast Cancer

PET imaging relies on positron emitting to provide information on biochemical, in vivo changes in many biological processes of various types of tumors. To date, [^18^F]FDG represents the radiopharmaceuticals most broadly used, as most malignant tumors overexpress glucose transporters and show increased hexokinase activity. In BC, FDG-PET is recommended for initial staging in patients with clinical stage ≥ IIB, while its use in clinical stage IIA is still debated. FDG-PET is also recommended in patients with suspected or known recurrence and for assessment of response to systemic treatments in metastatic BC patients [53,54].

However, FDG-PET holds limitations due to small lesion size (due to the low spatial resolution of PET tomographs and partial volume effect) or low FDG avidity. FDG uptake is influenced by the grade (according to the Elston–Ellis modification of the Scarff–Bloom-Richardson classification system), histological subtype, proliferation index (as assessed by the Ki67 index), hormone receptor status, and tumor phenotype. Indeed, grade 1–2 tumors, low-proliferative tumors, and hormone-positive ones show lower FDG uptake. Finally, FDG uptake is lower in ILC compared to IDC (namely for the primary lesion), and it is higher in HER2-positive and Triple Negative tumors rather than in Luminal subtypes (with Luminal A showing lower uptake compared to Luminal B) [55,56,57,58].

## 4. PET Imaging in ILC

### 4.1. [^18^F]Fluorodeoxyglucose-PET

Evidence about the diagnostic accuracy of FDG-PET in ILC is limited, as the majority of studies were designed in IDC. In a study considering one of the largest cohorts published, 146 newly diagnosed stage I-III ILC patients demonstrated a lower impact of FDG-PET on systemic staging for ILC than for IDC patients. PET scans were performed before systemic or radiation therapy, and unsuspected distant metastases, confirmed by biopsy, were found in 12 patients (8%), 2 of 50 stage II and 10 of 88 stage III. In three cases, patients were upstaged only by CT, as the lesions were not FDG-avid. In comparison, in a cohort of IDC patients with similar characteristics, patients were upstaged to the IV stage in 22% of cases [59]. Furthermore, FDG uptake was higher in untreated bone metastases of IDC patients than in ILC patients. Non-FDG-avid sclerotic bone metastases were more common in ILC patients compared to IDC and mixed ductal/lobular histology; therefore, CT images of the PET/CT scan should be carefully evaluated in ILC [60].

A retrospective analysis of patients with biopsy-proven BC with low FDG avidity demonstrated the usefulness of FDG-PET for the surveillance of advanced-stage ILC. A total of 491 PET scans of 192 patients with ILC, mucinous carcinoma, and tubular carcinoma were evaluated. A total of 142 scans were performed for initial staging (84 in ILC) and 349 as follow-up (186 scans in 89 ILC patients). Among 15 patients with stage IIB ILC, only one developed an ovary localization (6.7%), while six out of nine stage IIIC patients (66.7%) had recurrent disease detected by FDG-PET [61]. In another series of 24 ILC patients who performed 49 scans, the authors demonstrated high sensitivity and specificity for FDG-PET to evaluate the presence of metastatic disease. Survival rates significantly differ among patients with true negative and true positive PET scans, with a higher rate of death events in the latter [62]. A possible correlation between FDG uptake and prognosis has also been hypothesized by Fujii et al. [63], as authors evaluated preoperatively 196 patients with ILC (15 patients) and IDC (181 patients). Among ILC, a significant association was observed between FDG uptake in the primary lesion (evaluated as standardized uptake value—SUVmax) and tumor size and between SUVmax and nuclear grade, thus suggesting that FDG-PET might be a predictor of more aggressive ILC disease.

In the literature, data on FDG-uptake of ILCs rely predominantly on the evaluation of primary tumors, but differences in the hexokinase activity, other glycolytic enzymes, and biomarkers expression have been highlighted in BC [64,65] and may explain the discrepancies observed. As a highly heterogeneous disease, FDG-PET might be used to identify patients who are more likely to develop a disease with less favorable outcomes [66].

However, the current level of evidence does not allow us to draw definitive conclusions on the real impact of FDG-PET in ILC (Table 1).

### 4.2. [^18^F]Fluoroestradiol-PET

The over-expression of ER can be investigated with dedicated PET radiopharmaceuticals targeting the ER. The most successful ER imaging radiopharmaceutical is the [^18^F]16α-17β-fluoroestradiol ([^18^F]FES), approved by the US Food and Drug Administration in 2020. Several studies demonstrated the efficacy of ([^18^F]FES-PET/CT (FES-PET) for imaging evaluation of ER+ invasive BC, with high concordance between ER status determined by immunohistochemical methods and FES-PET results [67]. These studies evaluated both ILC and IDC together, with a large majority of cases comprising the latter. Therefore, there are no conclusive data about the efficacy of FES-PET in investigating ILC.

Nevertheless, FES-PET showed promising results as some authors evaluated retrospectively metastatic BC patients from six prospective trials using FES-PET conducted at the Memorial Sloan Kettering Cancer Center from 2008 to 2019 [68]. Among 92 patients enrolled, 14 were affected by ILC. Seven patients performed both FES- and FDG-PET within five weeks and were included in the retrospective evaluation. FES-PET and FDG-PET detected a total of 254 and 111 lesions, respectively. In five out of seven patients, FES-PET detected more lesions, with a more favorable tumor-to-background ratio. In one patient, only FDG-PET was able to detect liver metastases [68]. Another case series included three patients with suspected ILC recurrence. In comparison with radiological imaging and FDG-PET, FES-PET provided conclusive results that guided clinical decision-making and subsequent treatments [69]. Accordingly, there is great potential for this ER-targeted PET imaging, as FES-PET could provide a qualitative and quantitative assessment of multiple tumor sites simultaneously and could also evaluate sites that would be challenging to reach with biopsy. Moreover, the evaluation of hormonal receptor heterogeneity between the primary tumor and metastases or among different metastatic sites could guide the therapeutic management of patients, thus allowing for predicted response to endocrine therapy. In this setting, FES-PET could provide a non-invasive, in vivo measurement of the ligand binding function of ER in situ and could be helpful in the design of personalized therapeutic approaches. The main limitation of FES-PET could be its accuracy in detecting liver metastases due to tracer metabolization and, hence, the high physiological uptake of FES in the liver [70,71]. However, visual analysis associated with correction for background demonstrated to improve the diagnostic accuracy of FES-PET in patients with BC metastases to the liver, resulting in 83% specificity and 77% sensitivity [72].

### 4.3. Fibroblast Activation Protein Inhibitors-PET

Over the past decades, strong evidence supported the hypothesis that the complexity of cancer is related to the characteristics of tumor cells, together with the crosstalk between malignant cells and the different components of the tumor microenvironment (TME). TME is a heterogeneous and highly dynamic system composed of fibroblasts, immune cells, endothelial cells, precursor cells, signaling molecules, and extracellular matrix (ECM) components, which interact closely with tumor cells. It may account for up to 90% of the tumor mass in common malignancy as stomach, breast, and pancreatic carcinomas [73].

Key elements in TME are cancer-associated fibroblasts (CAFs), metabolically active, spindle-shaped cells with enhanced migratory and proliferative properties that release different proinflammatory cytokines and growth factors and play a crucial role in cancer cell invasion, growth and migration, immunosuppression, metabolic reprogramming, and angiogenesis [74,75].

CAFs represent the most abundant cell type of BC TME and constitute a heterogeneous population divided into four groups (CAF-S1 to CAF-S4) according to differential activation marker expression of α-smooth muscle actin (ASMA), fibroblast activation protein (FAP), platelet-derived growth factor (PDGF) receptor β, and CD29 [76]. FAP expression is high in CAFs and low in normal fibroblasts and, as a consequence, in normal adult human tissues. This differential expression made FAP a promising target for molecular imaging of a large variety of tumors and led to the development of FAP-targeting radiopharmaceuticals based on FAP-specific inhibitors (FAPis), such as [^68^Ga]FAPi-02, [^68^Ga]FAPi-04, and [^68^Ga]FAPi-46. These radiotracers bind to the enzymatic domain of FAP with very high specificity, and biodistribution studies on tumor-bearing mice and on a mixed population of different cancers demonstrated high intratumoral uptake of the radiopharmaceuticals and fast renal clearance. Moreover, these studies showed very low uptake in normal organs such as the liver, intestine, brain, and spine and, as a consequence, a higher tumor-to-background ratio (TBR), improving the diagnostic performance of PET imaging [77,78].

FAP demonstrated abundant expression in stroma across all BC molecular subtypes and higher expression both in tumoral and stromal cells of ILCs than IDCs [79,80]. However, the experience with FAPi-PET/CT is still very limited in BC and consists of heterogeneous series that enrolled few patients [81,82]. A retrospective study included 48 BC patients with local, locally advanced, and metastatic diseases. Five patients had ILC, and [^68^Ga]FAPi-04 PET/CT detected more lesions (mainly at the breast, lymph nodes, and bone), with higher uptake values compared to [^18^F]FDG PET/CT. Higher SUVmax was found with FAPi-PET compared to FDG-PET for ILC patients: uptake ratio of [^18^F]FDG and [^68^Ga]FAPI were indeed 0.39 (±0.19) and 0.24 (±0.12) for IDC and ILC, respectively, but these results did not reach statistical significance (*p* = 0.084) [82].

Thus, no strong data about the diagnostic accuracy of FAPi-PET/CT in ILBs are available, but the favorable biodistribution of FAP-specific inhibitors makes these agents particularly suitable for imaging these tumors with unique growth patterns and metastatic spread. Moreover, FAP-targeted molecules can also be labeled with high-energy isotopes for TME radio-ligand therapy, whose potential feasibility has already been highlighted in the first human studies. Radio-ligand therapy with [^90^Y]FAPI-46, [^177^Lu]FAPI-46, and [^177^Lu]FAP-2286 was well tolerated, with low radiation doses to non-target tissues, including the kidneys, and acceptable side effects (predominantly hematological) [78]. In conclusion, this promising field appears worthy of further investigation through targeted studies.

### 4.4. PET with Other Radiopharmaceuticals

Several other radiopharmaceuticals have been proposed for BC assessment, such as [^18^F]labeled 1-amino-3-fluorocyclo-butane-1-carboxylic acid ([^18^F]FACBC or [^18^F]fluciclovine) and [^64^Cu]Sarcophagine-Bombesin ([^64^Cu]SAR-BBN) have shown initial but interesting results in ILC.

[^18^F]fluciclovine is a synthetic amino acid, a leucine analog, that was developed for L-amino acid transport evaluation. The amino acid transporter ASCT2 is the major mediator of its uptake, with LAT1 and the system A amino acid transporter type 2 also contributing to cellular uptake [83]. [^18^F]fluciclovine is approved by the FDA and the European Commission for the detection of prostate cancer in patients with biochemical recurrence following prior definitive treatment and can also be used for PET/CT imaging in BC. Indeed, BC demonstrated increased protein synthesis associated with increased amino acid consumption and overexpression of amino acid transporters in the cell membrane [84].

Studies have shown that [^18^F]fluciclovine uptake is higher in BC than in benign lesions and healthy breast tissue, with higher uptake in patients with higher tumor grades. This tracer allowed the detection of metastases in the brain, bone, lung, and lymph nodes, while the high hepatic uptake limited the evaluation of liver lesions [85,86]. In a study enrolling 14 patients, both [^18^F]FDG PET/CT and [^18^F]fluciclovine PET were performed [86]. The four patients with ILC showed higher [^18^F]fluciclovine avidity than [^18^F]FDG avidity in the primary tumors (median SUVmax 6.1 vs. 3.7, respectively), while for the remaining 10 patients with IDC, there was an inverse relationship (median SUVmax 6.8 vs. 10.0, respectively) [86]. These data highlight the different metabolic processes measured by the two tracers and, given the initial encouraging results, could lead to further targeted studies in ILCs.

[^64^Cu]SAR-BBN is a gastrin-releasing peptide receptor (GRPR) antagonist conjugated to a sarcophagine derivative and radiolabeled with copper64. GRPR belongs to subtype II of the bombesin receptor (BBN) family, and its overexpression has been observed in BC, both in IDC and ILC [87]. A recent study included seven patients with metastatic hormone-positive/HER2 negative BC that performed both [^18^F]FDG and [^64^Cu]SAR-BBN PET/CT for re-staging purposes. Two of the seven patients had classical ILC, and images showed greater uptake and tumor volume on [^64^Cu]SAR-BBN PET/CT compared to FDG PET/CT (SUVmax 20 vs. 11 and 20 vs. <3) [88]. GRPR-positive BC with GRPR radioligands is a promising target for radioligand therapy as well, especially in ER-positive BC patients. Considering the emerging role of GRPR radio antagonists, antagonists demonstrated higher biosafety and attractive pharmacokinetics in animal models and humans compared to agonists, and clinical trials to assess the efficacy of the theranostic approach for GRPR-expressing tumors are expected soon [89].

## 5. Conclusions

The level of evidence about the role of PET imaging in ILC is still limited, despite the global impact of this specific disease pattern. Data regarding glucose metabolism have been mainly derived from studies enrolling both IDC and ILC. Moreover, the metabolic characteristics of the primary tumors were considered valid also for metastatic disease and did not account for the intra-patient heterogeneity of the disease. However, FDG-PET might be proposed as a prognostic biomarker to identify patients with less favorable outcomes. Recently, promising results have been observed with non-FDG radiopharmaceuticals highlighting the potential of the in vivo evaluation of the expression of the ER, FAP, and GRPR, as well as the amino acid metabolism. Therefore, even if the real clinical impact of FDG-PET in ILC is still unknown, there is still space for improvement in PET imaging, considering the promising results obtained by the new non-FDG radiopharmaceuticals. Nevertheless, clinical trials with larger and homogenous populations designed to test the diagnostic accuracy of these new diagnostic techniques are needed, with particular emphasis on the metastatic setting and the comparison among the different radiopharmaceuticals.

## Figures and Tables

**Table 1:** Overview of studies that specifically evaluated 18F-FDG PET/CT in patients with invasive lobular cancer.

18F-FDG PET/CT IN INVASIVE LOBULAR CARCINOMA					
Authors	N of Patients/Scan	Patients Characteristics	Clinical Setting	Study Design	Results
Hogan et al. [59]	146 ILC (comparison cohort: 89 IDC)	Newly diagnosed stage I-III ILC	Staging	R	8% ILC pts upstaged to IV vs. 22% of comparison cohort of stage III IDC
Dashevsky et al. [60]	13 ILC (+74 IDC and eight mixed ductal/lobular pts)	Newly diagnosed stage IV BC pts with bone metastases	Staging	R	Higher FDG uptake in untreated bone metastases of IDC pts vs. ILC pts
Park et al. [61]	192 pts (491 scans) 270 scans in ILC pts (84 stagings, 186 follow-ups)	Low FDG-avid BC (ILC, MC, and TC)	Staging and follow-up	R	Usefulness of FDG-PET for the surveillance of advanced stage ILC (detection of recurrence in 66.7% of stage IIIC ILC pts)
Orevi et al. [62]	24 pts (49 scans)	Histologically proven ILC (eight newly diagnosed)	Staging (eight scans) and follow-up (41 scans)	R	High sensitivity and specificity in evaluation of metastatic ILC (30/31 true positive results)
Fujii et al. [63]	15 ILC (+181 IDC)	Newly diagnosed BC pts	Staging	R	Linear association between SUVmax and ILC tumor size and nuclear grade

BC, breast cancer; ILC, invasive lobular cancer; IDC, invasive ductal cancer; MC, mucinous carcinoma; TC, tubular carcinoma; RF, radiopharmaceutical; FDG, 18F-fluoro-2-deoxy-D-glucose; SUV, standardized uptake value; R, retrospective.

## Data Availability

Not applicable.
